# Problematic internet use in children and adolescents: associations with psychiatric disorders and impairment

**DOI:** 10.1186/s12888-020-02640-x

**Published:** 2020-05-27

**Authors:** Anita Restrepo, Tohar Scheininger, Jon Clucas, Lindsay Alexander, Giovanni A. Salum, Kathy Georgiades, Diana Paksarian, Kathleen R. Merikangas, Michael P. Milham

**Affiliations:** 1 Healthy Brain Network, Child Mind Institute, New York, NY USA; 2 MATTER Lab, Child Mind Institute, New York, NY USA; 3 Department of Psychiatry, Universidade Federal do Rio Grande do Sul, Porto Alegre, Brazil; 4 Department of Psychiatry and Behavioural Neurosciences, McMaster University, Hamilton, Canada; 5 Genetic Epidemiology Research Branch, Intramural Research Program, National Institute of Mental Health, Bethesda, MD USA; 6 Center for the Developing Brain, Child Mind Institute, New York, NY USA; 7 Center for Biomedical Imaging and Neuromodulation, Nathan S. Kline Institute for Psychiatric Research, Orangeburg, New York, USA; 8 Child Mind Institute, New York, NY USA

**Keywords:** Internet addiction, Pediatric, Depression, ADHD, ASD, Impairment

## Abstract

**Background:**

Problematic internet use (PIU) is an increasingly worrisome issue, as youth population studies are establishing links with internalizing and externalizing problems. There is a need for a better understanding of psychiatric diagnostic profiles associated with this issue, as well as its unique contributions to impairment. Here, we leveraged the ongoing, large-scale Child Mind Institute Healthy Brain Network, a transdiagnostic self-referred, community sample of children and adolescents (ages 5–21), to examine the associations between PIU and psychopathology, general impairment, physical health and sleep disturbances.

**Methods:**

A total sample of 564 (190 female) participants between the ages of 7–15 (mean = 10.80, SD = 2.16), along with their parents/guardians, completed diagnostic interviews with clinicians, answered a wide range of self-report (SR) and parent-report (PR) questionnaires, including the Internet Addiction Test (IAT) and underwent physical testing as part of the Healthy Brain Network protocol.

**Results:**

PIU was positively associated with depressive disorders (SR: aOR = 2.43, CI: 1.22–4.74, *p* = .01; PR: aOR = 2.56, CI: 1.31–5.05, p = .01), the combined presentation of ADHD (SR: aOR = 1.91, CI: 1.14–3.22, p = .01; PR: n.s.), Autism Spectrum Disorder (SR: n.s.; PR: aOR = 2.24, CI: 1.34–3.73, *p* < .001), greater levels of impairment (SR: Standardized Beta = 4.63, CI: 3.06–6.20, p < .001; PR: Standardized Beta = 5.05, CI: 3.67–6.42, p < .001) and increased sleep disturbances (SR: Standardized Beta = 3.15, CI: 0.71–5.59, p = .01; PR: Standardized Beta = 3.55, CI: 1.34–5.75, p < .001), even when accounting for demographic covariates and psychiatric comorbidity.

**Conclusions:**

The association between PIU and psychopathology, as well as its impact on impairment and sleep disturbances, highlight the urgent need to gain an understanding of mechanisms in order to inform public health recommendations on internet use in U.S. youth.

## Background

The terms “problematic internet use”, “internet addiction”, “compulsive internet use” and “pathological internet use” have been used to refer to patterns of problematic behavior associated with internet use. Regardless of the specific terminology employed, concerns about the potential harms of internet use are growing rapidly [1–6], particularly in light of the inclusion of gaming disorder as a formal condition in the ICD-11 and as a condition requiring further study in the DSM-5. Here, we use the term problematic internet use (PIU) as a neutral alternative that does not inherently imply the presence of psychopathology. There is a consensus that PIU is characterized by overuse of the internet with associated impairment(s) across various domains of functioning [1–4, 7, 8]. Some have gone further, suggesting that PIU is a behavioral version of a substance-use disorder [9–11], while others have suggested that it is either an impulse-control disorder [3, 12, 13], or a subtype of obsessive-compulsive disorder [14, 15], although empirical evidence for these designations is lacking [8, 10, 16–20]. Regardless of whether PIU truly constitutes a form of addiction, there is a need to obtain a more complete empirical understanding of the contexts in which it arises in childhood and adolescence, as well as its unique contributions to socio-emotional and behavioral functioning.

Prevalence estimates of PIU typically range between 1 and 10%, although some estimates such as those in Asia have exceeded 25% [3, 21–30]. Accumulating evidence has documented associations between PIU and the presence of one or more mental disorders [4, 31, 32]. Depression has been consistently linked to PIU across studies measuring general internet use, smartphone use, and online gaming [6, 7, 26, 31–54]. Similarly, anxiety disorders, particularly social anxiety [26, 37, 43, 44, 47, 55, 56] and Generalized Anxiety Disorder (GAD) [37, 50, 54], have also been associated with PIU [6, 31, 38–40, 42, 45, 46, 48, 49, 51, 53]. PIU has been further shown to correlate with different components of anxious thought (i.e. fear of missing out, fear of negative evaluation) in the context of social anxiety [57]. Though generally less consistent, associations between PIU and Attention Deficit Hyperactivity Disorders (ADHD) [7, 26, 27, 37, 43, 45, 50, 58, 59], Autism Spectrum Disorders (ASD) [60–62], personality disorders [12, 37], obsessive-compulsive disorder [7, 31, 37, 46], and either schizophrenia, psychotic symptoms or dissociative symptoms [7, 37, 46, 48], have also been documented.

PIU has also been associated with impairment in various life domains, including problems with social relationships [4, 7, 30, 43, 63–65] and professional endeavors [7, 12]. PIU has also been linked to lower reported life satisfaction and well-being [40, 43, 66, 67]. Consistent with the range of findings reported, individuals who sought online mental health help had higher scores on a broad impairment scale [68]. PIU has also been associated with impairment in physiological and physical domains. For example, decreased physical fitness has been associated with PIU [49, 69–73] and physical activity was found to be a protective factor in the relationship between PIU and psychopathology [49, 74]. Additionally, there are links between PIU and sleep disturbances [36, 41, 50, 75], insomnia [42, 76], and lower sleep quality [39, 53, 76]. The structure of the relationship between PIU, poor sleep, physical health and psychopathology is unclear, as some studies have suggested mediating or moderating effects amongst the different variables [36, 39, 53]. It is unclear whether the impairment associated with PIU - both physiological and non-physiological - is independent from that caused by potentially comorbid psychological disorders present in individuals with PIU [33, 36, 53, 71, 72].

While many prior studies have separately found associations between PIU and impairment and PIU and psychiatric disorders, the present work explores both multidiagnostic assessments and general impairment in a single, diverse sample of youth. Additionally, measures include both parent and self-reports, allowing for comparison between informants. Here, we leveraged the ongoing, large-scale Child Mind Institute Healthy Brain Network, a transdiagnostic self-referred community sample of children and adolescents (ages 5–21), to examine the associations between PIU and psychopathology. The aims were: 1) to examine associations between PIU and clinician diagnoses of a range of mental disorders in youth controlling for the effects of comorbidity; and 2) to assess whether PIU was associated with general impairment and a reduction in health behaviors including poor physical health and sleep disturbances.

## Methods

### Sample/design

Participants were recruited from the ongoing Healthy Brain Network (HBN) initiative that seeks to create and share a 10,000-participant biobank of data from children and adolescents ages 5–21 from the New York City area [77]. Data collected includes psychiatric, cognitive, behavioral, genetic, and lifestyle information as well as MRI and EEG neuroimaging. The HBN has two main collection sites — one located on Staten Island and one in Midtown Manhattan. Participants were excluded from participation if there were outstanding safety concerns, insufficient verbal abilities, an IQ lower than 66, and neurological concerns that could interfere with MRI and EEG interpretation. Participants were asked to suspend stimulant medication meant for treatment of ADHD on the days of testing to minimize potential confounds, though participants who did not suspend medication use were still included in the study and medication use was noted on the day of assessment.

As part of the HBN survey battery, participants (SR) and their parents/guardians (PR) completed a variety of age-based questionnaires assessing basic demographic characteristics, dimensional assessments of domains associated with mental health, substance use, socioeconomic status and internet use. Information about the participant’s race was collected from parent verbal report during a structured clinical history interview. For participants under the age of 11, a trained research assistant read and explained individual items and collected responses from participants.

### Measures

#### Schedule for Affective Disorders and Schizophrenia - Children’s version (K-SADS)

Participants and their parents/guardians were independently administered the Schedule for Affective Disorders and Schizophrenia - Children’s Version (K-SADS) [78] by a trained member of the clinical team. For participants under the age of 11, clinicians determined whether the K-SADS would be administered to both child and parent or just parent based on verbal function and expected ability to tolerate the interview. Lifetime consensus diagnoses were based on DSM-5 diagnostic criteria using information from both the parent and child along with historical records and other HBN assessments [77] when clarifications were needed.

#### Internet Addiction Test (IAT)

Young’s IAT is a 20-item questionnaire ranked on a five-point Likert-type scale [79]. Both parent and self-reported versions were used to assess PIU. The total score resulted from summing all 20 items on the questionnaire, resulting in possible scores ranging from 0 to 100. The internal consistency was adequate in the current sample with a cronbach’s alpha value of 0.91 for self-report and 0.95 for parent-report. Additionally, cronbach’s alpha scores for the self-reported IAT did not differ substantially when calculated separately for participants below (alpha = 0.91) and above (alpha = 0.89) the age of 11. Based on previous work [9, 45, 80], a total sum score of 40 or above was considered Problematic Internet Use (PIU) and participants were then categorized as either non-problematic (absence of PIU) or problematic internet users (presence of PIU).

#### The Barratt Simplified Measure of Social Status (BSMSS)

The BSMSS is a parent-reported measure of social status, which is used as a proxy for socioeconomic status (SES) [81]. The total sum score used in the current report is calculated by adding the scores for the total occupation and total education subscales, which are answered in regards to both guardians. For participants with a single caregiver, total scores were calculated based solely on that caregiver’s responses. Total scores were then divided into tertiles to determine low, middle and high SES, respectively, for the demographics analysis and included as a continuous covariate for the rest. Since the presence or absence of a secondary caregiver explained a large amount of the partial missing data in the BSMSS, an additional binary variable representing this factor was also included as a covariate in all analyses.

#### Columbia Impairment Scale (CIS)

The CIS is a 13-item scale that assesses global functioning in domains of interpersonal relations, psychopathology, school performance, and use of leisure time [82]. The total sum score of all items was used for both self and parent-report versions. Internal consistency was adequate in the current sample (cronbach’s alpha for parent-report was 0.85 and 0.81 for self-report). Additionally, cronbach’s alphas on the self-reported CIS did not substantially differ when calculated separately for children below (alpha = 0.78) and above (alpha = 0.89) age 11.

#### Physical Activity Questionnaire (PAQ)

The PAQ is a self-report questionnaire asking participants to recall their physical activity levels in various domains for the past 7 days [83]. The PAQ-C is administered to children ages 8–14 and the PAQ-A, modified by removing items referring to school recess, is administered to ages 14–19. The total score is created using the average of all items after the first question, which are all rated on a scale from one to five. Cronbach’s alpha was 0.88 for the PAQ-C and 0.87 for the PAQ-A.

#### Sleep Disturbances Scale (SDSC)

The SDSC is a parent-reported 27-item questionnaire rated on a 5-point Likert type scale that assesses sleep disturbances in various domains (initiating and maintaining sleep, breathing, disorders of arousal, sleep-wake transition, excessive somnolence, and sleep hyperhidrosis) [84]. The total sum score of all items was used in the current report. Cronbach’s alpha in the current sample was 0.82.

#### Body composition measures

Participant height and weight were also collected by a trained research assistant in order to calculate Body Mass Index (BMI). Electric impedance and reactance measures were collected using electrodes placed at four positions on the body: two on the right hand and two on the right foot. RJL Systems Bio-Impedance Analysis was used to calculate Fat Mass Index (FMI).

### Analyses

Primary analyses were carried out using each, self and parent-report measures. Participants and parents who did not complete one or more of the questionnaires required for the analyses were removed from the sample. Due to the small number of individual missing items in the remaining sample, complete cases analysis was used. All analyses were also run using the maximum number of complete datasets for each (thus resulting in differential sample sizes for each analysis) in order to determine whether results differed when analyses were run using the final sample (see Tables S3-S6). The final sample consisted of 374 males and 190 females (total *N* = 564) participants between the ages of 7–15 (mean = 10.80, SD = 2.16) out of the original sample of 2094 children aged 5–21. Regarding those excluded (*n* = 1530), 1310 were missing data due to key questionnaires included in the present work being introduced later in the protocol’s timeline, and the remaining 220 were missing a combination of demographic information, physical activity information, and/or KSADS administration. All statistical analyses were conducted using The R Project for Statistical Computing for Windows [85] (version 3.5.2).

To examine demographic correlates of PIU, unadjusted prevalence odds ratios were calculated using the R package epiR [86]. Next, logistic regression was used to estimate three sets of odds ratios (ORs) for associations between mental disorders and PIU, with PIU as the outcome: unadjusted, adjusted for demographics (age, sex, SES, site, single caregiver, and race), and additionally adjusted for comorbid mental disorders. Demographics were included in the second and third sets of models to account for possible associations with mental disorders. Third, linear regression was used to assess the relationship between PIU and impairment, with impairment as the outcome. We again estimated three sets of models: unadjusted, adjusted for demographics, and further adjusted for mental disorders. Finally, we estimated associations of PIU with physical health and sleep disturbances using linear regression. Again, we first estimated unadjusted associations, followed by adjustment for demographics, and finally adding adjustment for mental disorders.

## Results

### Demographics

**Fig. 1:**
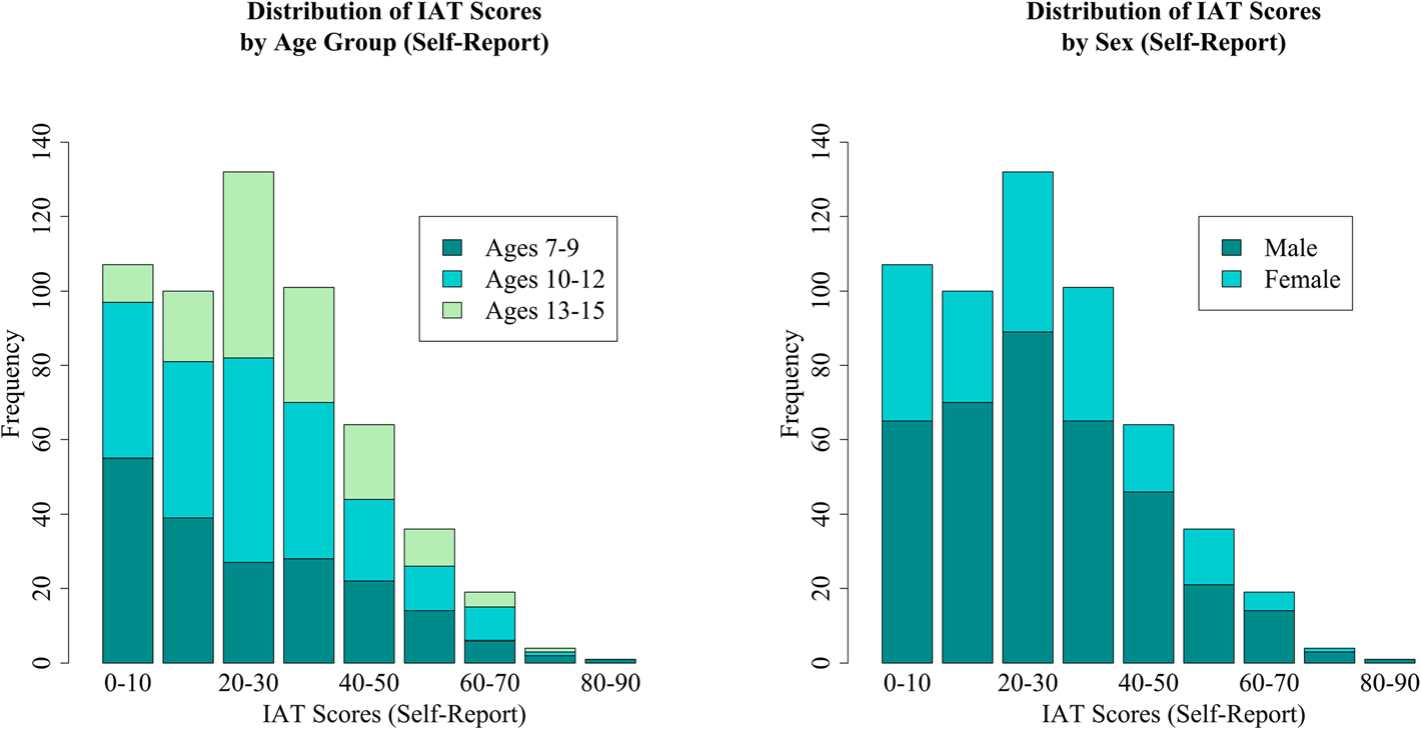
Distribution of Internet Addiction Test Scores (Self-Report) Self-reported Internet Addiction Test (IAT) score distributions broken down by participant age group and sex

**Fig. 2:**
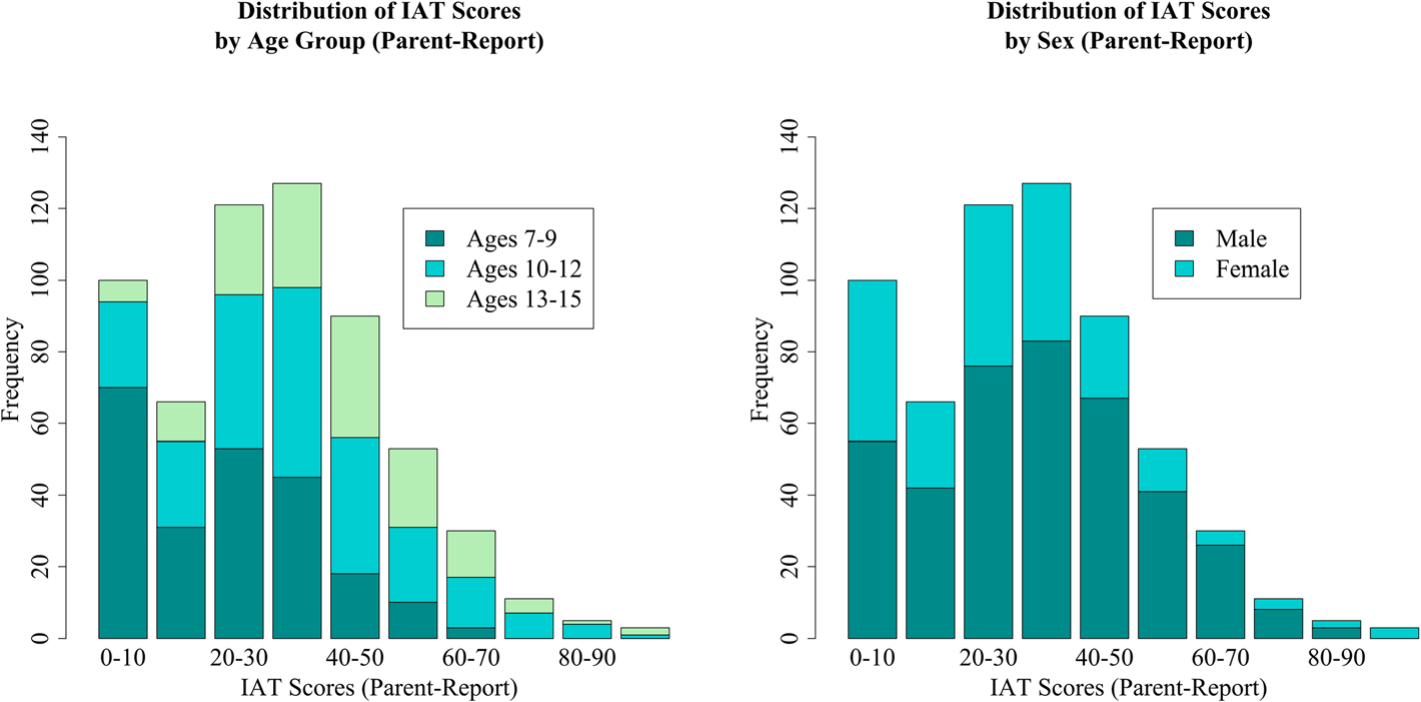
Distribution of Internet Addiction Test Scores (Parent-Report) Parent-reported Internet Addiction Test (IAT) score distributions broken down by participant age group and sex

Out of the total 564 participants (374 males and 190 females), 440 (78.01%) and 124 (21.99%) scored below and above the PIU cut-off, respectively. Although there were no significant differences in PIU by any of the demographic characteristics, PIU was more common in males than females, in those ages 10–12, and those in the high SES range (Table 1). See Figs. 1 and 2 for the distribution of IAT scores across age groups and sex for self and parent-report, respectively.

**Table 1:** Participant Demographics and Internet Use

	PIU Absent ***n*** = 440 (78.01%)	PIU Present ***n*** = 124 (21.99%)	OR (95% CI)	***p***
**Sex**				
Male	289 [51.24%]	85 [15.07%]	1.00 (ref)	–
Female	151 [26.77%]	39 [6.91%]	0.88 (0.57–1.34)	0.55
**Age**				
7–9	149 [26.42%]	45 [7.98%]	1.00 (ref)	–
10–12	181 [32.09%]	44 [7.8%]	0.80 (0.50–1.28)	0.36
13–15	110 [19.50%]	35 [6.21%]	1.05 (0.64–1.74)	0.84
**SES**				
Low SES	52 [9.22%]	14 [2.48%]	1.00 (ref)	–
Middle SES	85 [15.07%]	38 [6.74%]	1.66 (0.83–3.33)	0.16
High SES	303 [53.72%]	72 [12.77%]	0.88 (0.47–1.67)	0.70
**Race**				
Caucasian	240 [42.55%]	58 [10.28%]	1.00 (ref)	–
African American	52 [9.22%]	21 [3.72%]	1.67 (0.94–2.98)	0.08
Hispanic	43 [7.62%]	16 [2.84%]	1.54 (0.82–2.91)	0.19
Asian	11 [1.95%]	3 [0.53%]	1.13 (0.33–3.90)	0.86
Other Race	94 [16.67%]	26 [4.61%]	1.14 (0.68–1.92)	0.61
**Site**				
Staten Island	243 [43.09%]	72 [12.77%]	1.00 (ref)	–
Midtown	197 [34.93%]	52 [9.22%]	0.89 (0.60–1.33)	0.57

Number of participants within each demographic category who scored below and above 40 on the Internet Addiction Test; unadjusted odds ratios for each category

### Mental disorders

Tables 2 and 3 show the rates and odds ratios for associations between PIU and psychiatric disorders for self and parent-report, respectively. After adjustment for age, sex, SES, site, single caregiver, race and all other diagnoses of interest, there were significant positive associations between PIU and depressive disorders, regardless of reporter (SR: aOR = 2.43, CI: 1.22–4.74, *p* = .01; PR: aOR = 2.56, CI: 1.31–5.05, p = .01). Additional significant positive associations were found between: 1) self-reported PIU and the combined subtype of ADHD (SR: aOR = 1.91, CI: 1.14–3.22, p = .01; PR: n.s.) and, 2) parent-reported PIU and ASD (SR: n.s.; PR: aOR = 2.24, CI: 1.34–3.73, *p* < .001).

**Table 2:** Rates of Psychiatric Diagnosis with Internet Use (Self-Report)

Diagnosis	PIU Absent ***n*** = 440 (78.01%)	PIU Present ***n*** = 124 (21.99%)	OR (95% CI)	***p***	aOR^**a**^ (95% CI)	***p***	aOR^**b**^ (95% CI)	***p***
ASD	61 [70.93%]	25 [29.07%]	1.57 (0.94–2.62)	0.08	1.55 (0.90–2.61)	0.10	1.44 (0.83–2.46)	0.18
Anxiety	140 [76.50%]	43 [23.50%]	1.14 (0.75–1.73)	0.55	1.18 (0.76–1.82)	0.45	1.01 (0.60–1.68)	0.96
Depression	31 [62.00%]	19 [38.00%]	2.39 (1.30–4.37)	<.001	2.43 (1.26–4.61)	0.01	2.43 (1.22–4.74)	0.01
ADHD-C	107 [69.93%]	46 [30.07%]	1.84 (1.20–2.80)	<.001	1.97 (1.26–3.09)	<.001	1.91 (1.14–3.22)	0.01
ADHD-I	140 [79.55%]	36 [20.45%]	0.88 (0.57–1.35)	0.55	0.82 (0.52–1.28)	0.40	1.07 (0.63–1.78)	0.81
Social Anxiety	49 [77.78%]	14 [22.22%]	1.02 (0.55–1.90)	0.96	1.04 (0.53–1.94)	0.90	0.96 (0.43–2.04)	0.91

^**a**^Adjusted for sex, age, SES, collection site, and single caregiver; ^**b**^Adjusted for sex, age, SES, collection site, single caregiver and all diagnoses

Prevalence rates and adjusted odds ratios (95% CI) for associations between DSM-5 disorders (lifetime prevalence) and Problematic Internet Use (PIU) determined by a self-report score above or below a cut-off score of 40 on the Internet Addiction Test

**Table 3:** Rates of Psychiatric Diagnosis with Internet Use (Parent-Report)

Diagnosis	OR (95% CI)	***p***	aOR^**a**^ (95% CI)	***p***	aOR^**b**^ (95% CI)	***p***
ASD	2.25 (1.42–3.55)	<.001	2.28 (1.38–3.76)	<.001	2.24 (1.34–3.73)	<.001
Anxiety	1.19 (0.83–1.71)	0.34	1.13 (0.75–1.68)	0.56	1.18 (0.73–1.89)	0.50
Depression	3.16 (1.77–5.72)	<.001	2.34 (1.24–4.48)	0.01	2.56 (1.31–5.05)	0.01
ADHD-C	1.22 (0.83–1.78)	0.30	1.53 (1.00–2.33)	0.05	1.55 (0.95–2.52)	0.08
ADHD-I	1.40 (0.97–2.02)	0.07	1.18 (0.79–1.75)	0.43	1.41 (0.89–2.24)	0.14
Social Anxiety	1.00 (0.57–1.72)	0.99	0.69 (0.37–1.25)	0.23	0.51 (0.24–1.07)	0.08

^**a**^Adjusted for sex, age, SES, collection site, and single caregiver; ^**b**^Adjusted for sex, age, SES, collection site, single caregiver and all diagnoses

Prevalence rates and adjusted odds ratios (95% CI) for associations between DSM-5 disorders (lifetime prevalence) and Problematic Internet Use (PIU) determined by a parent-report score above or below a cut-off score of 40 on the Internet Addiction Test

### Indices of impact of PIU

Tables 4 and 5 present associations between PIU and each of the following: impairment assessed by self and parent-reported CIS scores, physical fitness assessed by self-reported PAQ scores, BMI, FMI, and sleep disturbances assessed by parent-reported SDSC scores. After adjustment for demographic factors and psychiatric disorders, PIU was associated with greater levels of impairment for both self- and parent-report (SR: Standardized Beta = 4.63, CI: 3.06–6.20, *p* < .001; PR: Standardized Beta = 5.05, CI: 3.67–6.42, p < .001). Although BMI was positively associated with PIU in unadjusted models, no significant differences emerged after adjustment for demographic and psychiatric disorders (SR: Standardized Beta = 0.61, CI: − 0.30-1.51, *p* = .19; PR: Standardized Beta = − 0.48, CI: − 1.30-0.34, *p* = .25). There was no significant association between PIU and our indicator of physical fitness or FMI (see Tables 4 and 5). PIU was significantly associated with sleep disturbances in the adjusted models (SR: Standardized Beta = 3.15, CI: 0.71–5.59, *p* = .01; PR: Standardized Beta = 3.55, CI: 1.34–5.75, p < .001).

**Table 4:** Multivariable Regressions for Negative Consequences of Internet Use (Self-Report)

Outcome	Model 1^**a**^ Beta (95% CI)	***p***	Model 2^**b**^ Beta (95% CI)	***p***	Model 3^**c**^ Beta (95% CI)	***p***
Impairment	5.63 (4.03–7.23)	<.001	5.37 (3.78–6.96)	<.001	4.63 (3.06–6.20)	<.001
Physical Activity	−0.03 (−0.19–0.13)	0.72	−0.03 (− 0.19–0.12)	0.67	−0.01 (− 0.16–0.15)	0.95
BMI	1.03 (0.08–1.99)	0.03	0.82 (−0.08–1.72)	0.07	0.61 (− 0.30–1.51)	0.19
FMI	0.64 (−0.02–1.31)	0.06	0.56 (− 0.07–1.19)	0.08	0.40 (− 0.24–1.03)	0.22
Sleep Disturbances	4.67 (2.14–7.20)	<.001	4.64 (2.10–7.17)	<.001	3.15 (0.71–5.59)	0.01

^**a**^Unadjusted model; ^**b**^Adjusted for sex, age, SES, collection site, and single caregiver; ^**c**^Adjusted for sex, age, SES, collection site, single caregiver and all diagnoses; Beta values are standardized

Separate linear regression models examining associations between self-reported Problematic Internet Use (PIU) and impairment, physical health, and sleep

**Table 5:** Multivariable Regressions for Negative Consequences of Internet Use (Parent-Report)

Outcome	Model 1^**a**^ Beta (95% CI)	***p***	Model 2^**b**^ Beta (95% CI)	***p***	Model 3^**c**^ Beta (95% CI)	***p***
Impairment	6.53 (5.09–7.97)	<.001	6.56 (5.03–8.09)	<.001	5.05 (3.67–6.42)	<.001
Physical Activity	−0.06 (− 0.20–0.07)	0.38	−0.04 (− 0.19–0.10)	0.56	0.00 (− 0.14–0.15)	0.98
BMI	0.96 (0.15–1.77)	0.02	− 0.18 (− 0.99–0.62)	0.65	−0.48 (−1.30–0.34)	0.25
FMI	0.39 (−0.17–0.95)	0.18	− 0.11 (− 0.68–0.46)	0.70	−0.32 (− 0.89–0.25)	0.27
Sleep Disturbances	4.51 (2.36–6.65)	<.001	4.78 (2.50–7.07)	<.001	3.55 (1.34–5.75)	<.001

^**a**^Unadjusted model; ^**b**^Adjusted for sex, age, SES, collection site, and single caregiver; ^**c**^Adjusted for sex, age, SES, collection site, single caregiver and all diagnoses; Beta values are standardized

Separate linear regression models examining associations between parent-reported Problematic Internet Use (PIU) and impairment, physical health, and sleep

### Supplementary materials

The IAT threshold score for differentiating between problematic and non-problematic internet use in our primary analyses was set to 40 based on the previous literature [9, 45, 80]. Supplementary analyses assessed the dependence of our odds ratio findings on the specific threshold employed. While adjusted PIU odds ratios did not remain significant at thresholds of 50 or higher for self-report, they were found to be more robust for parent-report, with odds ratios for depressive disorders remaining significant until the threshold was increased to 90, and ADHD-C until the threshold was increased to 70 (ASD was less robust, losing significance at 50 or higher).

Tables S1 and S2 provide a complementary perspective of PIU, in which the presence of problematic behaviors is treated dimensionally. This was accomplished by utilizing multiple regression to examine the relationship between IAT total scores (self and parent report, respectively), as opposed to the dichotomous PIU variable, and psychopathology. Consistent with our categorical findings, IAT total scores were significantly associated with: 1) both depression and ADHD-C, regardless of whether looking at self or parent report, and 2) ASD for parent report. An additional significant relationship between Social Anxiety and IAT scores was observed in parent-report only. Tables S3-S6 replicated analyses for both self and parent report with the maximum sample available for each analysis. Since Tables 4 and 5 utilized the maximum sample available, no replications for these two analyses were completed. Results obtained using the final sample (*N* = 564) were consistently replicated (see Additional File 1).

## Discussion

The current study examined associations between PIU and the presence of mental health disorders, impairment and health behaviors in a large, pediatric and transdiagnostic, self-referred community sample. Across self- and informant-based reporting methods, we found significant PIU associations with depressive disorders, ADHD, combined presentation and ASD. Importantly, PIU independently predicted impairment, even after accounting for the contributions of comorbid DSM-5 disorders. Consistent with prior studies [36, 50, 53, 76], the presence of PIU was associated with increased sleep disturbances. Surprisingly, when compared with previous work [72–74], PIU was not consistently negatively correlated with physical fitness, though some results trended towards significance. This may be because some pediatric participants have more structured exercise and play times dictated by caregivers (e.g. Physical Education class, compulsory after-school sports, etc.) and thus the deleterious effects of PIU on physical fitness are prevented. Findings were generally convergent between self and parent reports, though PIU associations with ADHD, combined presentation were subthreshold for parent-report, and those for ASD were not detectable with self report. Overall, findings from the present study suggest that while internet use is not necessarily linked to psychopathology, PIU can be, and may be associated with impairment even after accounting for comorbid psychopathology.

While depressive disorders have been consistently linked to PIU, associations with ADHD have been less commonly found [39, 45, 51, 53, 87]. Caplan has suggested that depressed individuals tend to harbor negative views of their social competency, thus encouraging them to seek social encounters online, as these may be ‘easier’ to achieve since they allow for an increased flexibility in self-presentation through anonymity [88]. Ko et al. have argued that internet use, particularly online gaming, provides necessary immediate reward, rapid response rates, and a plethora of activities to keep boredom at bay, thus making it highly appealing to individuals with ADHD [32]. Additionally, poor self-control and high impulsivity also increase the risk of developing PIU in individuals with ADHD [32]. Finally, in accordance with previous literature [60–62], associations with ASD were found, though solely for parent report, possibly suggesting a limitation in self awareness. The discrepancy in these parent-child reports, as well as the impact of ADHD comorbidity with ASD, are potential avenues for future research to elucidate ASD associations. Our findings have specific implications for the treatment of these disorders and the recognition of PIU, and may provide a pathway to understanding possible mechanisms for the maintenance of these disorders.

We did not confirm findings from earlier studies of an association between PIU with anxiety disorders and symptoms of anxiety [47, 50, 55, 57]. Researchers have theorized that individuals with anxiety, particularly social anxiety, use online relationships to compensate for poor real-life ones in a similar way as posited for individuals suffering from depression [32, 55]. However, many of these previous studies did not account for comorbidity with ADHD, ASD and depression. Therefore, prior associations between PIU and anxiety may have been attributable to comorbid conditions.

In accordance with previous literature [36, 41, 42, 50, 53, 75, 76], we found an association between PIU and sleep problems, adding to the growing awareness of the impact of inadequate sleep in U.S. adolescents on both mental and physical health [89, 90]. The persistence of this association after adjustment for not only demographic variables, but also ADHD and depression, suggests that internet use may directly influence sleep behavior or vice versa. This suggests that future studies should assess the direct impact of internet use, particularly problematic use, on sleep.

Finally, our findings imply that the effects of PIU on impairment are independent of the effects of comorbid psychopathology on impairment. The potential clinical implications suggest that PIU may not only be problematic, but also may lead to serious impairment, especially for individuals who already suffer from a comorbid mental health disorder**.** Recent changes to the DSM-5 and ICD-11 have also highlighted the importance of providing more information on negative consequences of specific internet-related activities (i.e. sedentary behavior and sleep deprivation), and how these may contribute to impairment in various domains of life, particularly in samples at risk for psychiatric disorders. Future research should provide more information on the specific negative consequences of different internet-related activities (i.e. sedentary behavior and sleep deprivation), and how these may contribute to impairment in various domains of life, particularly in populations at risk for psychiatric disorders.

There were several limitations to the current work. First, due to the community self-referred nature of the sample, our results may be affected by sampling biases, which can decrease their generalizability to the larger population of youths in the United States. Our sample does have a higher representation of males, likely due to higher frequency of disruptive behaviors, which tend to be among the most common reasons families seek help. Additionally, there is also a greater representation of children from higher SES and Caucasian youth, which we controlled for in our analyses. Importantly, the convergence of our findings with prior studies that have established associations between PIU and psychiatric symptomatology using other sampling designs (i.e. samples not recruited in a clinical setting) [39, 45, 51, 53, 60–62, 87] decreases the likelihood that our results can be substantively explained by the properties of our particular sample. Nonetheless, replication of our findings in more representative samples would ensure their robustness. Second, PIU was only assessed through one questionnaire which, though demonstrated to be reliable, lacks specificity. The questionnaire does not specify what activities “internet use” and “being online” refer to, leaving interpretation to the respondent; it also has no mention of social media or smartphone usage, reflecting the timing of its creation. The Internet Process Addiction Test (IPAT) [91] has been developed to address these issues, though is notably longer and yet to be heavily adopted in the literature. The present work used a general measure of PIU, which is able to capture problematic behavioral patterns associated with internet use, regardless of the specific activities an individual is engaging in. In future work, the acquisition of activity-specific measures would be helpful in discerning differences in patterns of association between mental health disorders and specific forms of internet addiction (e.g., video-gaming, pornography, gambling). Third, while parent-reported PIU was available for children of all ages in the sample (5–21), self-reported questionnaires were only administered to children ages 7 and up. Fourth, the HBN sample is largely composed of individuals affected by one or more mental health or learning disorders, inherently increasing the likelihood of problematic behaviors among participants. As such, the odds ratios calculated for disorders such as ADHD and depression may be an underestimate of what would be obtained in a more traditional, community representative sample. Finally, the sample size of the present work was not large enough to enable stable odds ratio estimates based on typically developing children alone.

Future research should focus on identifying the specific internet-related activities (i.e. social media, gaming, pornography access, gambling, etc. [2].) that are associated with different clinical disorders and comorbidity, as preliminary work has determined that different activities may have different relationships with psychopathology, behaviors, and personality characteristics [56, 57, 87, 92–94]. Most importantly, potential mechanisms through which PIU is associated with impairment and sleep problems after adjustment for psychiatric disorders requires further inquiry. Identification of the directionality of these relationships should be pursued in prospective research in order to generate effective interventions to prevent the negative outcomes associated with PIU.

## Conclusions

The present study provides empirical evidence for links between PIU and depressive disorders, Attention Deficit Hyperactivity Disorder and Autism Spectrum Disorders in a large community sample of youth. The present study highlights the importance of exploring PIU in younger samples due to the myriad of negative behaviors associated with this construct. Further research is necessary to identify potential mechanisms for these associations and their impact on general functioning and sleep. It also presents options for interventions in order to weaken not only the occurrence of these negative behaviors but also the strength of the relationship between them.

## Supplementary information

**Additional file 1.** Contains tables of complementary analyses.

## Data Availability

The datasets generated and/or analysed during the current study are available in the Child Mind Institute Healthy Brain Network repository, http://fcon_1000.projects.nitrc.org/indi/cmi_healthy_brain_network/

## Abbreviations

PIU: Problematic internet use
IAT: Internet addiction test
SR: Self-report
PR: Parent-report
