# Supplementary material for: Problematic internet use in children and adolescents: associations with psychiatric disorders and impairment
Source: BMC Psychiatry. 2020 May 27;20:252. doi: 10.1186/s12888-020-02640-x (PMC7251845; doi:10.1186/s12888-020-02640-x)
Supplement: Supplementary file 1 — Additional file 1. Contains tables of complementary analyses. [file 12888_2020_2640_MOESM1_ESM.docx]

**TABLE S1** Multiple linear regressions with clinical diagnoses (lifetime prevalence) predicting numerical Internet Addiction Test total scores for self-report (N = 564).

| **Predictor** | **Model 1^a^ B (95% CI)** | ***p*** | **Model 2^b^ B (95% CI)** | ***p*** | **Model 3^c^ B (95% CI)** | ***p*** |
| --- | --- | --- | --- | --- | --- | --- |
| ASD | 0.62 (-3.33-4.58) | 0.76 | 0.04 (-3.91-3.98) | 0.99 | -0.56 (-4.52-3.40) | 0.78 |
| Anxiety | 1.13 (-1.90-4.17) | 0.46 | 1.03 (-2.01-4.07) | 0.51 | -0.10 (-3.45-3.66) | 0.95 |
| Depression | 8.15 (3.20-12.11) | <.001 | 6.14 (1.07-11.21) | 0.02 | 5.89 (0.71-11.08) | 0.03 |
| ADHD-C | 3.78 (0.59-6.96) | 0.02 | 4.65 (1.42-7.88) | <.001 | 4.60 (0.98-8.22) | 0.01 |
| ADHD-I | -0.83 (-3.90-2.24) | 0.60 | -1.67 (-4.74-1.41) | 0.29 | 0.02 (-3.35-3.40) | 0.99 |
| Social Anxiety | 0.78 (-3.74-5.29) | 0.73 | 0.26 (-4.24-4.77) | 0.91 | -0.09 (-5.33-5.15) | 0.97 |
| ***Note:*** ***^a^****Unadjusted model;* ***^b^****Adjusted for sex, age, SES, collection site, and single caregiver;* **^c^***Adjusted for sex, age, SES, collection site, single caregiver and all diagnoses.* | | | | | | |

**TABLE S2** Multiple linear regressions with clinical diagnoses (lifetime prevalence) predicting numerical Internet Addiction Test total scores for parent-report (N = 605).

| **Predictor** | **Model 1^a^ B (95% CI)** | ***p*** | **Model 2^b^ B (95% CI)** | ***p*** | **Model 3^c^ B (95% CI)** | ***p*** |
| --- | --- | --- | --- | --- | --- | --- |
| ASD | 6.36 (1.97-10.75) | <.001 | 5.11 (1.03-9.18) | 0.01 | 4.57 (0.51-8.63) | 0.03 |
| Anxiety | 1.33 (-2.03-4.70) | 0.44 | 0.22 (-2.92-3.36) | 0.89 | 0.96 (-2.66-4.58) | 0.60 |
| Depression | 12.80 (7.23-18.37) | <.001 | 7.49 (2.12-12.85) | 0.01 | 7.90 (2.49-13.32) | <.001 |
| ADHD-C | 2.85 (-0.69-6.39) | 0.11 | 4.36 (1.04-7.67) | 0.01 | 4.78 (1.12-8.43) | 0.01 |
| ADHD-I | 3.29 (-0.12-6.70) | 0.06 | 1.43 (-1.74-4.59) | 0.38 | 3.21 (-0.20-6.63) | 0.07 |
| Social Anxiety | -1.49 (-6.53-3.56) | 0.56 | -4.60 (-9.28-0.07) | 0.05 | -5.85 (-11.21-(-0.48)) | 0.03 |
| ***Note:*** ***^a^****Unadjusted model;* ***^b^****Adjusted for sex, age, SES, collection site, and single caregiver;* **^c^***Adjusted for sex, age, SES, collection site, single caregiver and all diagnoses.* | | | | | | |

**TABLE S3** Replication of Table 2 - adjusted odds ratios for self-reported Problematic Internet Use (PIU) for each diagnosis of interest (lifetime prevalence) - using the maximum sample available for the analysis (N = 927). Results were consistent with those from the limited sample.

| **Diagnosis** | **OR (95% CI)** | ***p*** | **aOR^a^ (95% CI)** | ***p*** | **aOR^b^ (95% CI)** | ***p*** |
| --- | --- | --- | --- | --- | --- | --- |
| ASD | 1.31 (0.88-1.92) | 0.17 | 1.25 (0.83-1.85) | 0.28 | 1.16 (0.77-1.74) | 0.47 |
| Anxiety | 1.19 (0.86-1.64) | 0.29 | 1.20 (0.85-1.67) | 0.29 | 1.13 (0.76-1.65) | 0.53 |
| Depression | 1.96 (1.25-3.02) | <.001 | 1.88 (1.15-3.02) | 0.01 | 1.86 (1.12-3.06) | 0.01 |
| ADHD-C | 1.58 (1.14-2.19) | 0.01 | 1.63 (1.15-2.30) | 0.01 | 1.66 (1.11-2.46) | 0.01 |
| ADHD-I | 0.97 (0.69-1.35) | 0.86 | 0.93 (0.66-1.31) | 0.69 | 1.17 (0.79-1.72) | 0.43 |
| Social Anxiety | 0.89 (0.51-1.48) | 0.65 | 0.86 (0.49-1.45) | 0.58 | 0.77 (0.41-1.41) | 0.41 |
| ***Note:*** ***^a^****Adjusted for sex, age, SES, collection site, and single caregiver;* ***^b^****Adjusted for sex, age, SES, collection site, single caregiver and all diagnoses.* | | | | | | |

**TABLE S4** Replication of Table 3 - adjusted odds ratios for parent-reported Problematic Internet Use (PIU) for each diagnosis of interest (lifetime prevalence) - using the maximum sample available for the analysis (N = 1362). Results for depression were consistent with those for the limited sample, and ADHD combined type emerged as significant (in the limited sample it only approached significance). While ASD was significant in both models for the limited sample, it remained significant only for the first model in the larger sample. Finally, ADHD inattentive type emerged as significant in the full sample while it did not even approach significance in the limited sample.

| **Diagnosis** | **OR (95% CI)** | ***p*** | **aOR^a^ (95% CI)** | ***p*** | **aOR^b^ (95% CI)** | ***p*** |
| --- | --- | --- | --- | --- | --- | --- |
| ASD | 1.52 (1.11-2.05) | 0.01 | 1.43 (1.02-2.01) | 0.04 | 1.32 (0.93-1.86) | 0.12 |
| Anxiety | 1.54 (1.18-2.00) | <.001 | 1.37 (1.01-1.84) | 0.04 | 1.31 (0.93-1.85) | 0.12 |
| Depression | 3.92 (2.63-5.85) | <.001 | 2.38 (1.51-3.76) | <.001 | 2.39 (1.49-3.84) | <.001 |
| ADHD-C | 1.28 (0.98-1.67) | 0.07 | 1.62 (1.20-2.19) | <.001 | 2.03 (1.43-2.88) | <.001 |
| ADHD-I | 1.89 (1.43-2.47) | <.001 | 1.41 (1.05-1.89) | 0.02 | 1.94 (1.38-2.73) | <.001 |
| Social Anxiety | 1.33 (0.85-2.02) | 0.20 | 0.98 (0.60-1.56) | 0.92 | 0.78 (0.44-1.34) | 0.37 |
| ***Note:*** ***^a^****Adjusted for sex, age, SES, collection site, and single caregiver;* ***^b^****Adjusted for sex, age, SES, collection site, single caregiver and all diagnoses.* | | | | | | |

**TABLE S5** Replication of Table S1 - three multiple linear regression models with the presence or absence of each diagnosis of interest (lifetime prevalence) predicting self-reported Internet Addiction Test total scores - using the maximum sample available for the analysis (N = 927). Results were consistent with those from the limited sample.

| **Predictor** | **Model 1^a^ B (95% CI)** | ***p*** | **Model 2^b^ B (95% CI)** | ***p*** | **Model 3^c^ B (95% CI)** | ***p*** |
| --- | --- | --- | --- | --- | --- | --- |
| ASD | 1.24 (-2.79-3.26) | 0.88 | -0.38 (-3.37-2.61) | 0.80 | -0.89 (-3.92-2.14) | 0.56 |
| Anxiety | 1.50 (-0.92-3.92) | 0.22 | 1.24 (-1.19-3.66) | 0.32 | 0.93 (-1.86-3.72) | 0.51 |
| Depression | 7.46 (3.87-11.06) | <.001 | 4.51 (0.74-8.27) | 0.02 | 4.25 (0.40-8.10) | 0.03 |
| ADHD-C | 2.73 (0.21-5.26) | 0.03 | 3.45 (0.89-6.00) | 0.01 | 3.39 (0.53-6.25) | 0.02 |
| ADHD-I | -0.64 (-3.13-1.85) | 0.61 | -1.29 (-3.73-1.16) | 0.30 | 0.13 (-2.54-2.79) | 0.93 |
| Social Anxiety | 0.00 (-3.85-3.85) | >.99 | -0.80 (-4.59-2.98) | 0.68 | -1.50 (-5.80-2.80) | 0.49 |
| ***Note:*** ***^a^****Unadjusted model;* ***^b^****Adjusted for sex, age, SES, collection site, and single caregiver;* **^c^***Adjusted for sex, age, SES, collection site, single caregiver and all questionnaires.* | | | | | | |

**TABLE S6** Replication of Table S2 - three multiple linear regression models with the presence or absence of each diagnosis of interest (lifetime prevalence) predicting parent-reported Internet Addiction Test total scores - using the maximum sample available for the analysis (N = 1362). Results for ASD, depression, ADHD combined type and social anxiety were consistent through both samples. Nonetheless, ADHD inattentive type emerged as significant in all three regression models in the large sample. Likewise, anxiety emerged as significant in the first and third regression models in the large sample.

| **Predictor** | **Model 1^a^ B (95% CI)** | ***p*** | **Model 2^b^ B (95% CI)** | ***p*** | **Model 3^c^ B (95% CI)** | ***p*** |
| --- | --- | --- | --- | --- | --- | --- |
| ASD | 4.32 (1.55-7.08) | <.001 | 3.31 (0.85-5.78) | 0.01 | 2.56 (0.09-5.03) | 0.04 |
| Anxiety | 4.11 (1.77-6.46) | <.001 | 1.94 (-0.19-4.06) | 0.07 | 2.78 (0.39-5.17) | 0.02 |
| Depression | 13.45 (9.62-17.29) | <.001 | 4.73 (1.08-8.37) | 0.01 | 4.90 (1.25-8.56) | 0.01 |
| ADHD-C | 2.04 (-0.28-4.37) | 0.09 | 3.87 (1.76-5.98) | <.001 | 4.90 (2.63-7.18) | <.001 |
| ADHD-I | 6.81 (4.37-9.25) | <.001 | 2.78 (0.58-4.99) | 0.01 | 4.86 (2.52-7.21) | <.001 |
| Social Anxiety | 0.66 (-3.25-4.56) | 0.74 | -2.93 (-6.40-0.53) | 0.10 | -4.93 (-8.80-(-1.06)) | 0.01 |
| ***Note:*** ***^a^****Unadjusted model;* ***^b^****Adjusted for sex, age, SES, collection site, and single caregiver;* **^c^***Adjusted for sex, age, SES, collection site, single caregiver and all questionnaires.* | | | | | | |
